# Curcumin Inhibits Age-Related Vascular Changes in Aged Mice Fed a High-Fat Diet

**DOI:** 10.3390/nu10101476

**Published:** 2018-10-10

**Authors:** Kenichiro Takano, Junko Tatebe, Naohiro Washizawa, Toshisuke Morita

**Affiliations:** 1 Department of Laboratory Medicine, Toho University Graduate School of Medicine, Tokyo 143-8540, Japan; takanoken@hotmail.com (K.T.); cvlabo@med.toho-u.ac.jp (J.T.); 2 Takano Hospital, Tokyo 144-0033, Japan; 3 Nutrition Therapy Center, Toho University Omori Medical Center, Tokyo 143-8541, Japan; washi@med.toho-u.ac.jp

**Keywords:** curcumin, high-fat diet, senescence, inflammation, heme oxygenase

## Abstract

Inhibiting the onset of arteriosclerotic disease, which has been increasing due to the westernized diet and aging, is a significant social challenge. Curcumin, a type of polyphenol, has anti-oxidative effects and anti-inflammatory action and is expected to treat and to have prophylactic effects on different diseases. In this study, we examined the effects of long-term administration of curcumin on vascular aging and chronic inflammation—the causes of arteriosclerotic disease. Eight-week-old C57BL/6J mice were fed with high fat diet (HFD) or 0.1% curcumin-mixed HFD (HFD + Cu) until 80 weeks old (*n* = 20 for each group). After the breeding, we examined the expression of antioxidant enzymes, heme oxygenase-1 (HO-1), oxidative stress, vascular aging, and inflammatory changes in the aorta. In the HFD group, oxidative stress increased with decreased sirt1 expression in the aorta followed by increased senescent cells and enhanced inflammation. Whereas in the HFD + Cu group, HO-1 was induced in the aorta with the suppression of oxidative stress. Additionally, it was shown that sirt1 expression in the aorta in the HFD + Cu group remained at a level comparable to that of the 8-week-old mice with suppression of increased senescent cells and enhanced inflammation. Consequently, disorders associated with HFD were resolved. These results suggest that curcumin might be a food with a prophylactic function against arteriosclerotic disease.

## 1. Introduction

Aging is a risk factor for arteriosclerotic disease, which is the same in Europe, America, and Japan. Different epidemiological studies have shown that the increase in the older age group is associated with an increased risk of arteriosclerotic disease [1,2,3]. Moreover, in Japan, fat intake has been increasing as the dietary pattern has become westernized. This increase in fat-derived energy intake has been reported as a possible risk factor for arteriosclerotic disease [4,5,6,7]. Obesity and glycolipid metabolism disorder induced by this increased fat intake trigger oxidative stress characterized by increased reactive oxygen species and decreased anti-oxidative function as well as inflammatory reaction associated with abnormal secretions of adiponectin and inflammatory cytokines, which leads to the onset of arteriosclerotic disease [8,9,10,11,12,13,14]. Since preventing the onset and inhibiting the recurrence of arteriosclerotic disease are significant social challenges in Japan—a country with a super-aging society and an increasingly westernized diet—the expectations and efficacy of different functional foods on disease prevention and treatment have been actively studied [15,16,17].

Curcumin, a main component of natural turmeric (*Curcuma longa* Linn, Turmeric), is a type of polyphenol, which has long been used for curry spice, Chinese traditional herbal medicine, or in Japan, as food coloring for Japanese confectioneries. In the United States, curcumin is approved as safe by the Food and Drug Administration (FDA) [18]. Recent studies, which have been gaining global attention, have revealed that curcumin has different physiological activities in the body. Curcumin is known to have anti-oxidative and anti-inflammatory actions, anticancer action associated with cytostasis, induction of apoptosis, and anti-angiogenesis, anti-virus action, and cytoprotective action [19,20,21,22]. Furthermore, curcumin is known to have hypoglycemiclipidemic effects, affecting various transcription factors that control gene expression involved in glucose and fat metabolism [23,24,25], and curcumin intake is expected to possibly reduce different disorders caused by a high-fat diet (HFD). However, it is unclear whether curcumin has beneficial effects on arteriosclerotic disease which is increasing in ordinary people who are on HFD and aging.

The anti-oxidative enzyme HO-1 is one of the mechanisms that mediates various actions of curcumin [26,27]. It metabolizes heme and produces iron ions, carbon monoxide, and biliverdin. Biliverdin is immediately converted to bilirubin by reductase, and has recently been reported to affect endothelial nitric oxide synthase (eNOS) and Sit1 expression [28,29,30,31]. HO-1 is thus known to exert anti-oxidation, anti-inflammation, immunoregulation, and anti-aging actions by a variety of mechanisms. According to earlier studies with mice on the effects of curcumin on vascular damage caused by HFD, curcumin exerts anti-arteriosclerotic action by affecting the inhibition of cholesterol absorption, the macrophage scavenger receptor expression, Toll-like receptor 4 (TLR4) expression, and the renin-angiotensin system [23,32,33,34,35,36].

Against this background, focusing on HO-1, we studied the effects of long-term curcumin intake on vascular damage in 8- to 80-week-old wild-type mice that are on HFD and are aging.

## 2. Materials and Methods

### 2.1. Reagent

Curcumin and Stannous Mesoporphyrin (SnMP) were purchased from Sigma-Aldrich (St Louis, MO, USA) and Frontier Scientific Inc. (Logan, UT, USA), respectively.

### 2.2. Animals

Male C57BL/6J (CLEA, Tokyo, Japan) mice were used for this study. All animal experiments were conducted after obtaining consent from Toho University Animal Care and User Committee (15-51-285) and in accordance with Toho University Guidelines for Animal Experiments. Six-week-old mice were bred in a breeding room at a room temperature of 25 °C and in a 12-h light–dark cycle of 8:00 to 20:00. Mice drank tap water ad libitum and were fed with standard feed (MF; Oriental Yeast, Tokyo, Japan). Two weeks later, 8-week-old mice (*n* = 80) were divided into four groups (*n* = 20 for each group), and until 80 weeks old, mice in the MF group were fed with standard feed; mice in the HFD group were fed with HFD (32% safflower oil, 33.1% casein, 0.5% DL-methionine, 17.6% sucrose, 1.4% vitamin mixture, 9.8% mineral mixture, 5.6% cellulose powder) [37,38]; mice in the HFD + Cu group were fed with HFD + 0.1% curcumin; mice in the HFD + Cu + SnMP group were fed with HFD + 0.1% curcumin + SnMP. Eight-week-old mice were in a young control group (y-CTL group). SnMP (10 μmol/Kg) was injected intraperitoneally once a week, mice were weighed weekly, and food intake was measured once every 4 weeks.

Upon completion of the experiment, we measured the blood pressure of mice followed by 16 h of fasting, and subsequently, blood samples of mice were collected under chloral hydrate anesthesia while they were sacrificed. Then, collection of aortas and various evaluations were performed. Blood samples were stored as blood serum at −80 °C for analysis. Blood pressure was measured with the tail-cuff method using a noninvasive automated sphygmomanometer (softron BP-98A: Softron Co., Ltd., Tokyo, Japan).

### 2.3. Superoxide Production

We produced fresh frozen sections of thoracic aortas of mice that were 80 weeks old upon completion of the experiment and of mice in the y-CTL group (*n* = 9 for each group). The frozen segments were incubated with dihydroethidium (DHE: 10 μmol/L in PBS) (Sigma-Aldrich, St Louis, MO, USA) in a light-shielded humidifier chamber at 37 °C for 30 min. Superoxide was detected as red florescence using ethidium [39]. A He-Ne laser at 543 nm was used in combination with a long-pass filter at 560 nm for the detection of ethidium bromide.

### 2.4. Measurement of Urinary 8-OHdG

Upon completion of the experiment, a 24-h collection of urine was performed using mouse metabolism cages (*n* = 9 for each group). The urine was centrifuged at 3000 rpm for 10 min, and subsequently supernatant was collected. Urinary 8-hydroxy-2′-deoxyguanosine (8-OHdG) was measured using an 8-OHdG ELISA kit (Japan Institute for the Control of Aging, Shizuoka, Japan) in accordance with the attached protocol [40].

### 2.5. Measurements of mRNA

Extraction of total RNA and Real-Time RT-PCR were conducted as previously reported [41]. ISOGEN (Nippon Gene, Toyama, Japan) was used for the extraction of total RNA from aorta. Monocyte chemoattractant protein-1 (MCP-1) mRNA was detected by real-time RT-PCR using an iQ5 Real-Time PCR Detection System (Bio-Rad, Hercules, CA, USA) and iScript One-Step RT-PCR Kit with SYBR Green (Bio-Rad, Hercules, CA, USA). PCR-primer sequences for MCP-1 were as follows: 5′-CAGCCAGATGCAGTTAACGC, 5′-GCCTACTCATTGGGATCATCTTG-3′ and GAPDH:5′-GCTGTGGTGGTGAAGCTGTA-3′, 5′-TGTTACCAACTGGGACGTCT-3′.

### 2.6. Western Blot Analysis

Western blot analysis was conducted as previously reported [41]. Aortas were homogenized in ice cooled lysis buffer (1% Triton X-100, 50 mM Hepes [pH 7.4], 100 mM sodium pyrophosphate, 100 mM sodium fluoride, 10 mM EDTA, 10 mM sodium vanadate, and protease inhibitor cocktail), followed by centrifugation at 15,000 rpm for 30 min at 4 °C and collection of supernatants (*n* = 5 for each group). Samples (2 to 10 μg) were resolved by 10% or 12.5% SDS polyacrylamide gel electrophoresis (SDS-PAGE) and transferred to a polyvinylidene difluoride (PVDF) membrane. Thereafter, the membrane was blotted with 5% skim milk for 1 h, reacted with various primary antibodies at 4 °C overnight, and reacted with secondary antibody on the following day. The samples were then visualized using Pierce Western Blotting Substrate Plus (Thermo Fisher Scientific Inc., Waltham, MA, USA). Images were obtained by the ChemiDoc XRS System (Bio-Rad, Hercules, CA, USA) and quantified by PDQuest software (Bio-Rad, Hercules, CA, USA). For the primary antibody, anti-HO-1 antibody (Stress Gen Biotechnologies Inc., Victoria, BC, Canada), Sirt1 antibody (Merck Millipore, Billerica, MA, USA), and β-actin antibody (Santa Cruz Biotechnology, Santa Cruz, CA, USA) were used, and for the secondary antibody, anti-rabbit IgG, HRP-linked whole antibody donkey (GE Healthcare, Buckinghamshire, UK) was used.

### 2.7. SA β-Gal Staining

Thoracic aortas were immediately fixed after being washed with ice-cooled PBS (*n* = 5 for each group). Subsequently, the aortas were incubated with senescence-associated β-galactosidase (SA-β-Gal) staining solution (pH 6) containing 1 mg/mL 5-bromo-4-chrolo-3-indlyl β-D-galactopylanoside (X-gal), 5 mmol/L potassium ferrocyanide, 5 mmol/L potassium fericyanide, 150 mmol/L NaCl, 2 mmol/L MgCl_2_, 0.01% sodium deoxycholate, and 0.02% Nonidet-40 at 37 °C for 24 h, followed by washing with PBS and detection of aged parts by blue color development [42].

### 2.8. Bilirubin Staining

After thoracic aortas were formalin-fixed and paraffin-embedded, the aortas were cut into 4 μM segments (*n* = 5 for each group). Immunohistochemical staining was performed after deparaffinization. For the primary antibody, anti-bilirubin antibody (Dojindo Laboratories, Kumamoto, Japan) was used, and for the secondary antibody and detection, a VECTOR M.O.M. Immunodetection Kit (Vector Laboratories, Inc., Burlingame, CA, USA) was used. Specimens were examined microscopically by light microscope (OLYMPUS BX50, Tokyo, Japan).

### 2.9. Serum Biochemical Analysis

Total serum bilirubin, total cholesterol, and blood glucose levels were measured by Nescoat VL T-BIL (Alfresa Pharma Co., Osaka, Japan), Determiner L TC II test kit (Kyowa Medex Co., Ltd, Tokyo, Japan), and GA09 (A&T Corporation, Tokyo, Japan), respectively. The measurements were performed with *n* = 10 for each group.

### 2.10. Measurement of MCP-1 Levels in Blood

MCP-1 in blood serum (*n* = 5 for each group) was measured using a Quantikine Mouse MCP-1 ELISA kit (R&D Systems, Minneapolis, MN, USA) in accordance with the attached protocol.

### 2.11. Statistical Test

All data were listed as mean ± standard error (standard error of mean: SEM). Group comparisons were performed by one-way ANOVA, and a *p*-value of <0.05 was considered statistically significant.

## 3. Results

### 3.1. Curcumin Controls Weight Gain and Increases the Blood Glucose Level and Blood Cholesterol Level Associated with Long-Term Administration HFD

**Figure 1:**
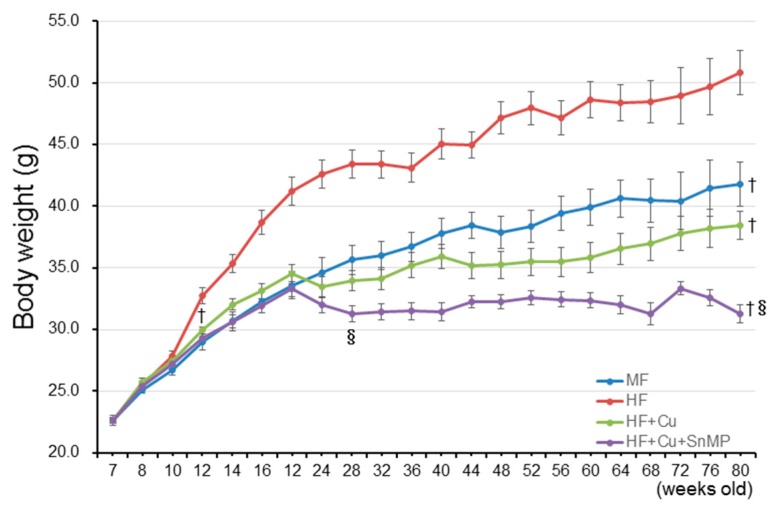
Mice were fed with standard feed (MF), high-fat diet (HFD), 0.1% curcumin-mixed HFD (HFD + Cu), or 0.1% curcumin-mixed HFD with administration of SnMP (HFD + Cu + SnMP) for 72 weeks from 8 weeks old to 80 weeks old. Data are presented in mean ± standard error for each group (*n* = 20). † *P* < 0.05. vs. HFD, § *P* < 0.05 vs. HFD + Cu. SnMP, Stannous Mesoporphyrin.

As shown in Table 1, there were no significant differences in the volume of feed intake between the other three groups even though the volume was low in the HFD + Cu + SnMP group. Next, the trends of BW in each group were evaluated. Compared with the MF group, BW in the HFD group significantly increased at 12 weeks old, which continued until 80 weeks old. Compared with the HFD group, weight gain was significantly suppressed at 12 weeks old in the HFD + Cu group, which continued until 80 weeks old. Compared with the HF+Cu group, weight gain was significantly suppressed at 28 weeks old in the HFD + Cu + SnMP group, which continued until 80 weeks old (Figure 1). There were no significant differences in systolic blood pressure (SBP) between all groups. Although the blood glucose level in the HFD group was significantly higher than in the MF group, the blood glucose level in the HFD + Cu group was significantly lower than in the HFD group. The blood glucose level in the HFD + Cu + SnMP group was significantly high compared with that of the HFD + Cu group, which, however, was significantly lower compared with that of the HFD group (Table 1). In the HFD group, the total cholesterol (TC) level in blood was significantly higher compared with that of the MF group; however, the TC level in the HFD + Cu group was significantly lower compared with that of the HFD group. The TC level in the HFD + Cu + SnMP group was comparable to that of the HFD + Cu group (Table 1).

### 3.2. Curcumin-Mixed Feed Enhances HO-1 Enzyme Activity

**Figure 2:**
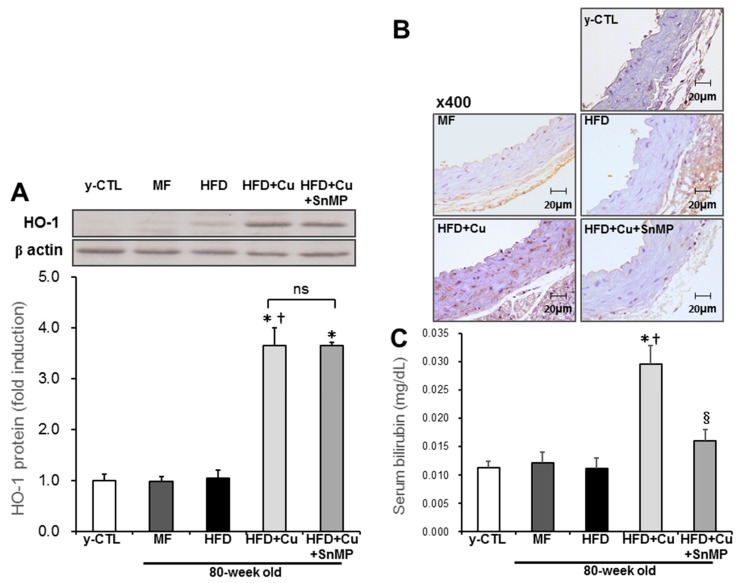
Effect of curcumin on HO-1 activity in mice. MF (standard feed), High-fat diet (HFD), 0.1% curcumin-mixed HFD (HFD + Cu), or 0.1% curcumin-mixed HFD plus SnMP (HFD + Cu + SnMP) was fed to mice for 72 weeks. (**A**) HO-1 expression in the aorta was assessed by Western blotting. The expression level was standardized by β-actin. (**B**) Bilirubin expression in the thoracic aorta was assessed by immunohistochemistry. (**C**) Blood bilirubin concentration was measured by a bilirubin assay kit. Data were presented in mean ± standard error for each group (*n* = 5). * *P* < 0.05 vs. y-CTL, † *P* < 0.05 vs. HFD, § *P* < 0.05 vs. HFD + Cu. SnMP, Stannous Mesoporphyrin; HO-1, Heme Oxygenase-1; y-CTL, 8 weeks old mice.

Previous reports showed that curcumin increases the expression of HO-1 [26,27]. We examined the expression of HO-1 in aortic tissue associated with curcumin-mixed HFD feed using the Western blot method. As shown in Figure 2A, HO-1 protein expression in the aorta in the HFD group and MF group were comparable to that of the y-CTL group; however, in the HFD + Cu group, HO-1 protein expression in the aorta significantly increased. Additionally, augmentation of HO-1 protein expression by curcumin was not affected in the HFD + Cu + SnMP group. Next, the enzyme activity of HO-1 was assessed by immunohistochemical staining of bilirubin expression in the aortic vessel wall and blood bilirubin concentration. As shown in Figure 2B, the assessment showed increased brown-dyed bilirubin expression mainly in aortic smooth muscle cells in the HFD-Cu group, whereas the curcumin effect disappeared in the HFD + Cu + SnMP group. Additionally, the blood bilirubin concentration significantly increased in the HFD-Cu group compared with the y-CTL group; however, no increase was noted in the blood bilirubin concentration in the HFD + Cu + SnMP group (Figure 2C). These results showed that curcumin enhances HO-1 enzyme activity.

### 3.3. Curcumin-Mixed Feed Suppresses Vascular and Systemic Oxidative Stress

**Figure 3:**
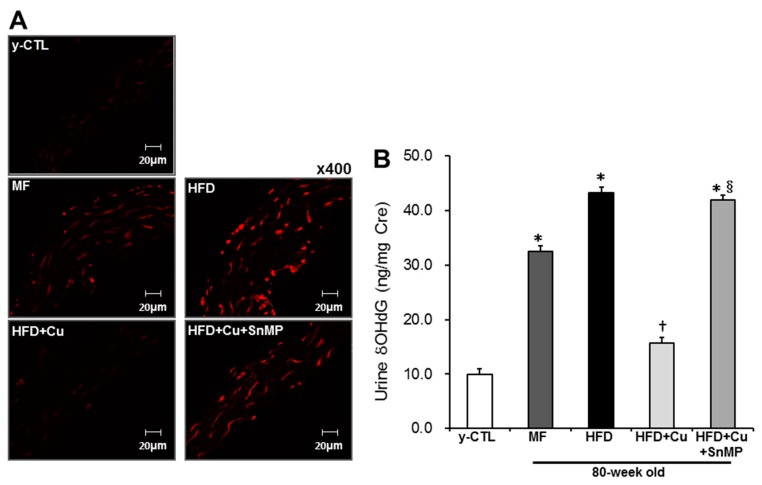
Effect of curcumin on oxidative stress in mice. MF (standard feed), High-fat diet (HFD), 0.1% curcumin-mixed HFD (HFD + Cu), or 0.1% curcumin-mixed HFD plus SnMP (HFD + Cu + SnMP) was fed to mice for 72 weeks. (**A**) The production of superoxide in the thoracic aorta was evaluated by DHE staining. (**B**) The urinary 8-OHdG concentration was measured using 8-OHdG ELISA kit. Data were presented in mean ± standard error for each group (*n* = 9). * *P* < 0.05 vs. y-CTL, † *P* < 0.05 vs. HFD, § *P* < 0.05 vs. HFD + Cu. SnMP, Stannous Mesoporphyrin; DHE, dihydroethidium; 8OHdG, 8-hydroxy-2′-deoxyguanosine; y-CTL, 8 weeks old mice.

Next, the anti-oxidative action of HO-1 was assessed, which had enhanced enzyme activity due to curcumin-mixed feed. Regional aortic oxidative stress was assessed with DHE staining of superoxide production in the vascular wall. As shown in Figure 3A, in the MF group, increased production of superoxide was noted in the vascular wall. This increase intensified more in the HFD group. However, in the HFD + Cu group, it was controlled to a level comparable to that of the y-CTL group. In the HFD + Cu + SnMP group, on the other hand, the curcumin-related inhibition effect on superoxide production disappeared. Systemic oxidative stress was evaluated with urinary 8OHdG concentration. Urinary 8OHdG in the MF group and HFD group significantly increased compared with that of the y-CTL group. This increased urinary 8OHdG in the HFD group, which was significantly higher compared with that of the MF group. There were no significant differences between the urinary 8OHdG in HFD + Cu group and the y-CTL group. However, the curcumin-related anti-oxidative effect had disappeared in the HFD + Cu + SnMP group (Figure 3B).

### 3.4. Enhanced HO-1 Activity Due to Curcumin-Mixed Feed Maintains Sirt1 Expression

**Figure 4:**
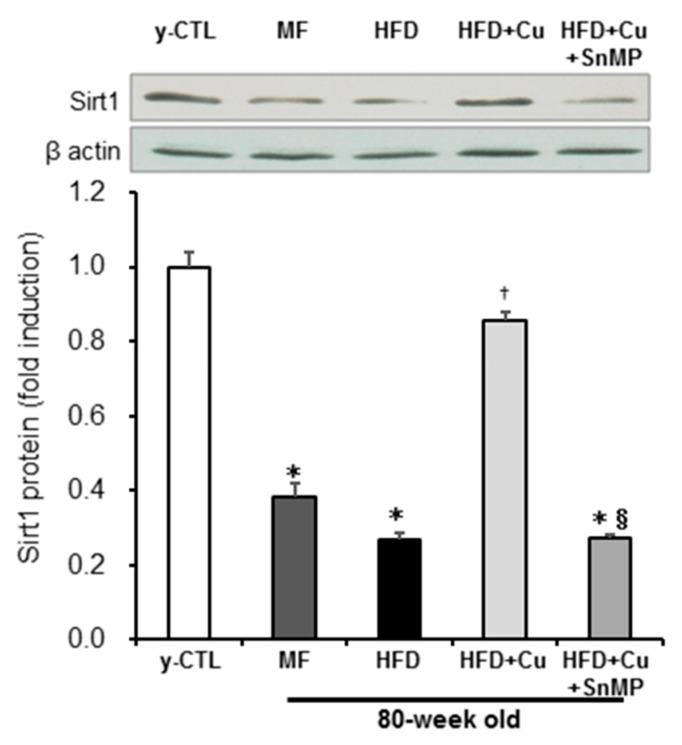
Effect of curcumin on Sirt1 expression in mice. MF (standard feed), High-fat diet (HFD), 0.1% curcumin-mixed HFD (HFD + Cu), or 0.1% curcumin-mixed HFD plus SnMP (HFD + Cu + SnMP) was fed to mice for 72 weeks. Sirt1 protein expression in the aorta was assessed by Western blotting. The expression level was standardized by β-actin. Data were presented in mean ± standard error for each group (*n* = 5). * *P* < 0.05 vs. y-CTL, † *P* < 0.05 vs. HFD, § *P* < 0.05 vs. HFD + Cu. SnMP, Stannous Mesoporphyrin; y-CTL, 8 weeks old mice.

While our previous studies revealed that increased ROS decreases intracellular NAD^+^, causes disturbance in Sirt1 activity, and promotes cellular aging [41,43,44], curcumin has been reported to suppress premature cellular aging in vascular endothelial cells via the activation of Sirt1 [45]. Sirt1 protein expression in the aorta was then assessed by Western blot analysis. The expression of Sirt1 protein in the MF group and HFD group significantly decreased compared with that of the y-CTL group. Moreover, this Sirt1 protein expression in the HFD group showed a sharper decline than in the MF group. However, Sirt1 protein expression in the HFD + Cu group remained at the same level as in the y-CTL group. These curcumin effects, however, disappeared in the HFD + Cu + SnMP group (Figure 4).

### 3.5. Curcumin-Mixed Feed Inhibits Chronic Inflammation Associated with Vascular Aging

**Figure 5:**
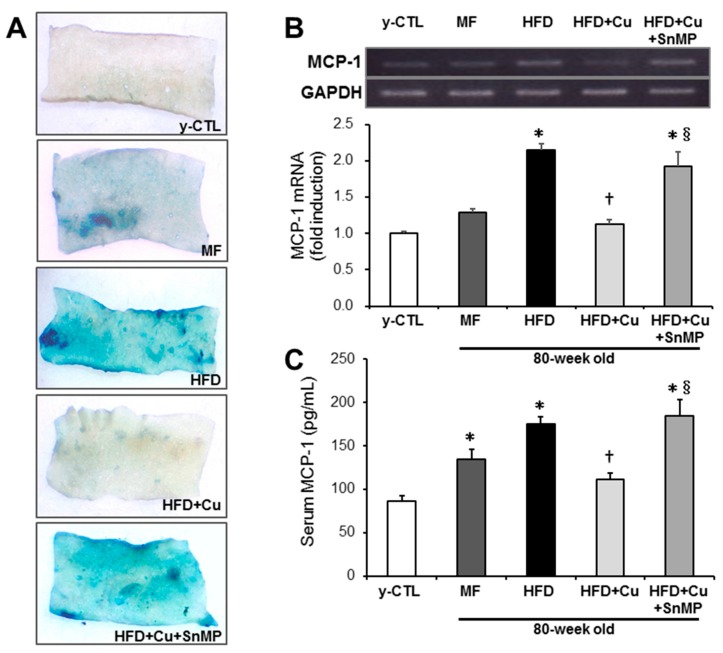
Effect of curcumin on SA β-gal activity and MCP-1 expression in mice. MF (standard feed), High-fat diet (HFD), 0.1% curcumin-mixed HFD (HFD + Cu), or 0.1% curcumin-mixed HFD plus SnMP (HFD + Cu + SnMP) was fed to mice for 72 weeks. (**A**) An increase in senescent cells in the thoracic aorta was analyzed by SA β-gal staining. (**B**) Expression of MCP-1 mRNA in the aorta was assessed by real-time RT-PCR. The expression level was standardized by GAPDH. (**C**) MCP-1 blood levels were measured by an MCP-1 ELISA kit. Data were presented in mean ± standard error for each group (*n* = 5). * *P* < 0.05 vs. y-CTL, † *P* < 0.05 vs. HFD, § *P* < 0.05 vs. HFD + Cu. SnMP, Stannous Mesoporphyrin; SA β-gal, senescence-associated β-galactosidase; y-CTL, 8 weeks old mice.

The accumulation of senescent cells and chronic inflammatory changes were assessed by SA-β-Gal staining and the expression of MCP-1, respectively. As shown in Figure 5A, compared with the y-CTL group, increased SA-βGal activity in the intima of thoracic aortas was noted in the MF group and HFD group. This increase was noted more intensively in the HFD group, which, however, in the HFD + Cu group, was controlled to the same level with the y-CTL group. These curcumin effects, however, disappeared in the HFD + Cu + SnMP group. Next, the expression of MCP-1 mRNA in the aorta was evaluated by real-time RT-PCR, and blood MCP-1 was assessed by ELISA. Although MCP-1 gene expression in the aorta significantly increased in the HFD group compared with the y-CTL group, the MF group showed no significant differences while presenting an increasing trend. By contrast, MCP-1 gene expression in the HFD + Cu group remained at the same level as in the y-CTL group. The blood MCP-1 level in the MF group and HFD group significantly increased compared with that in the y-CTL group. However, this blood MCP-1 level in the HFD group was significantly high compared with the MF group. The blood MCP-1 level in the HFD + Cu group showed no significant differences with the y-CTL group. In the HFD + Cu + SnMP group, the curcumin-related suppressive effect on MCP-1 mRNA expression in the aorta and MCP-1 blood level disappeared (Figure 5B,C).

## 4. Discussion

In the C57BL/6J group, while systemic and vascular oxidative stress increased due to 72 weeks of HFD, Sirt1 expression decreased and chronic inflammation progressed, and these changes were worse than in same-week-old mice in the MF group. It was revealed that curcumin-mixed feed suppresses these disorders, and that these anti-oxidative, anti-aging, and anti-inflammatory curcumin effects are possibly mediated by HO-1 activation.

It has become evident that aging and increased fat-derived energy intake are risk factors for arteriosclerotic disease [1,2,3,4,5,6,7]. Thus, in Japan—a country with an unprecedented aging society and where fat intake has been increasing due to the westernized diet—preventing the onset and suppressing the recurrence of arteriosclerotic disease are significant social challenges. In this study, the vascular effect of curcumin in an aging population on HFD was evaluated by using the following feeding groups: a group with C57BL/6J mice fed with HFD with a fat-derived calorie ratio (Fat kcal%) of approximately 60% of total energy for a long period of 72 weeks from 8 to 80 weeks old; a group fed with curcumin-mixed HFD feed.

C57BL/6J mice have been reported to be highly receptive to diet-induced obesity and thus become obese by HFD and develop hyperglycemia and hypercholesteremia [37,38]. Even in our study, although the volume of food intake in the HFD group was the same as that in the MF group, BW, blood glucose level, and TC significantly increased in the HFD group compared with the MF group. Curcumin intake is expected to possibly relieve glycolipidemic metabolic disorder caused by a high-fat diet [23,24,25], and in our study, BW, blood glucose level, and TC were also controlled to the same levels as the MF group by administration of curcumin-mixed feed. Additionally, the blood glucose level significantly increased with the administration of SnMP compared with the curcumin administration group; however, BW and TC were not affected and thus did not increase. Since it has been revealed that curcumin decreases the blood glucose level via HO-1 in diabetes and HFD mice [46,47], it is also suggested that in our study, the hypoglycemic action of curcumin is possibly involved in the HO-1 pathway. It is known that curcumin affects various transcription factors involved in lipid metabolism and becomes involved in the suppression of body weight gain and the improvement in lipid metabolism via many different mechanisms such as the proliferation of prelipocytes, the suppression of adipose cell differentiation, and fatty acid β oxidation [23,24,25,29,48,49]. In our study, since we did not evaluate adipose tissue, we have not revealed the detailed mechanism of suppressions of weight gain and an increase in TC by curcumin-mixed HFD. There were no significant differences in SBP among all groups, and SBP was the same as that of a regular C57BL/6J mouse. As has been reported, curcumin does not affect SBP at the level of normal control [50,51]. The number of 80-week-old mice alive in the curcumin administration group equaled the number of same-week-old mice in the MF group. Thus, it was suggested that we safely perform long-term administration of curcumin, starting at a young age. However, some clinical trials have shown that some anti-oxidative supplements possibly increase the risk of cancer [52,53,54]. Therefore, in order to conduct this study on curcumin’s effects in a clinical manner, we believeed that a sensitive approach was necessary with regard to administration volume and period.

Previous studies revealed that the expression of the anti-oxidative enzyme HO-1 increased, while curcumin suppresses acute hepatopathy and neonatal hypoxic-ischemic encephalopathy [26,27]. HO-1, one of the two HO isoforms, is expressed and induced in response to various stresses and produces CO of an equal moles to heme ratio, reduced iron (Fe^2+^), and biliverdin by using protoheme IX, a prosthetic group of heme protein, as a substrate. Each reactant has various actions. It has been reported that biliverdin becomes bilirubin by reductase, shows a very high anti-oxidative ability, and protects cells and tissues from oxidative damage [28,30,55]. Next, the expression of HO-1 in the aorta, associated with the administration of curcumin-mixed feed, was confirmed. Curcumin enhanced the expression of HO-1 protein in the mice aorta and increased bilirubin expression in aortic smooth muscle cells, a reaction product of the enzyme, with a significant increase in blood bilirubin concentration. The administration of SnMP, an inhibitor of HO-1 enzyme activity, significantly suppressed bilirubin expression in the aorta and blood bilirubin concentration. These results revealed that curcumin enhanced HO-1 enzyme activity. Subsequently, HO-1 anti-oxidative action with enhanced enzyme activity by curcumin was confirmed. Curcumin suppressed urinary 8OHdG and the production of superoxide in the vascular wall caused by the long-term administration of HFD at levels almost equal to those of the young control group. By contrast, these curcumin effects disappeared with the administration of SnMP. From all of the above, it was revealed that increased HO-1 enzyme activity is possibly associated with curcumin-related anti-oxidative effects.

Previous studies have reported that increased ROS decreases NAD^+^, creates interference with Sirt1 activity, and promotes cellular aging [41,43,44]. The reduced activity of Sirt1, a protein that belongs to NAD-dependent histone deacetylase, is reported to trigger aging-associated diseases [56]. Vascular endothelial cells with overexpression of Sirt1 have been revealed to be resistant to oxidative stress and to suppress the aging of vascular endothelial cells [57], and curcumin is reported to suppress premature cellular aging in vascular endothelial cells via Sirt1 activity [45]. Additionally, enhanced phosphorylation of eNOS due to HO-1 has been reported to suppress the aging of vascular endothelial cells [31], and it is suggested that not only Sirt1, but also eNOS might be deeply involved in anti-vascular aging associated with HO-1. Therefore, in this study, by focusing on Sirt1, we evaluated the effects of curcumin on Sirt1 expression in the aorta. The results showed that although Sirt1 protein expression in the aorta significantly decreased in the HFD group compared with the y-CTL group, Sirt1 protein expression in the HFD + Cu group remained comparable to that of the y-CTL group. This curcumin effect, however, disappeared due to the administration of SnMP. These facts showed that curcumin-related augmentation of Sirt1 expression is associated with anti-oxidative activity associated with HO-1 activation.

It was revealed that aging and increased fat-derived energy intake are risk factors for arteriosclerotic disease, and enhanced oxidative stress and chronic inflammation are considered as the cause of arteriosclerotic disease [8,9,10,11,58]. Chronic inflammation is likely to develop in elderly people, and it was revealed that inflammatory cytokine levels in the blood are high even in healthy individuals [59,60]. Additionally, it has been recognized that in healthy individuals, inflammatory cytokine levels in the blood increased due to the intake of food with a fat-derived energy ratio of 34% [7], and it has been reported that in animal experiments, HFD increases MCP-1 levels in the blood and subsequently triggers vascular inflammation [61]. Furthermore, an accumulation of senescent cells has been revealed to trigger inflammation [62,63], and lastly, SA-β-Gal staining was performed as a marker for cellular senescence. The expression of MCP-1 mRNA in the aorta and MCP-1 levels in the blood were assessed as markers for inflammatory changes. The results showed an SA-β-Gal-positive area in the aorta and significant increases in MCP-1 mRNA and MCP-1 blood levels in the HFD group while curcumin suppressed them to levels comparable to those of y-CTL. This curcumin effect, however, disappeared due to SnMP administration. These results revealed that long-term administration of curcumin to mice reduces oxidative stress via HO-1 activation and decreases the accumulation of senescent cells in the aorta while controlling inflammatory changes, and that not only Sirt1 but also phosphorylated eNOS [64,65] is possibly involved in this anti-aging effect of curcumin. By contrast, the effect of curcumin on the suppression of weight gain and the improvement of lipid metabolism did not disappear in spite of the administration of SnMP, which probably suggests that HO-1 activation is not heavily involved in this curcumin effect. Additionally, food intake in the SnMP group being significantly lower than that of the HFD + Cu group suggests adverse events due to long-term administration of SnMP. Thus, by inhibiting HO-1 with another technique, for instance, siRNA, the involvement of curcumin effects in HO-1 will be more clearly defined in terms of its effects on the improvement of obesity and glycolipid metabolism, anti-oxidation, anti-inflammation, and anti-vascular aging.

## 5. Conclusions

Curcumin is reported to have multifaceted benefits such as anti-cancer effects, anti-inflammatory action, and anti-oxidative action, and to have various physiological activities in vivo. This study revealed that curcumin suppresses vascular aging and inflammation, the causes of arteriosclerotic disease triggered by long-term administration of HFD. These results suggest that curcumin might be a food with a prophylactic function against arteriosclerotic disease. We believe that the potential of curcumin needs to be confirmed by clinical trials in the future.

## Figures and Tables

**Table 1:** Body weight, blood pressure, and blood glycolipid concentration of 80-week-old mice.

Parameters	y-CTL	MF	HFD	HFD + Cu	HFD + Cu + SnMP
Food intake (g/day)		4.2 ± 0.23	4.3 ± 0.23	4.2 ± 0.20	3.7 ± 0.13 §
Body weight (g)		41.8 ± 1.12 † (*n* = 18)	50.8 ± 1.80 (*n* = 14)	38.4 ± 1.20 † (*n* = 18)	31.3 ± 0.74 †§ (*n* = 12)
Systolic blood pressure (mmHg)	110 ± 4.3	113 ± 2.0	111 ± 42.6	106 ± 3.3	103 ± 8.7
Blood glucose (mg/dL)	119 ± 5.3	127 ± 4.2	211 ± 16.8 *	139 ± 6.0 *†	155 ± 8.6 *†§
Total cholesterol (mg/dL)	81 ± 7.1	91 ± 4.8	123 ± 11.9 *	99 ± 8.1 *†	103 ± 8.6 *

MF, fed with standard feed; HFD, high-fat diet; HFD + Cu, HFD with curcumin. () shows the number of mice alive upon completion of the experiment. SnMP, Stannous Mesoporphyrin. Values are mean ± SEM. * *P* < 0.05 vs. y-CTL, † *P* < 0.05 vs. HFD, § *P* < 0.05 vs. HFD + Cu.
